# Sex differences in rumen fermentation and microbiota of Tibetan goat

**DOI:** 10.1186/s12934-022-01783-8

**Published:** 2022-04-07

**Authors:** Xinyu Guo, Yuzhu Sha, Weibing Lv, Xiaoning Pu, Xiu Liu, Yuzhu Luo, Jiang Hu, Jiqing Wang, Shaobin Li, Zhidong Zhao

**Affiliations:** College of Animal Science and Technology/Gansu Key Laboratory of Herbivorous Animal Biotechnology, Gansu Agricultural University, Lanzhou, 730070 China

**Keywords:** Tibetan goat, Ruminal microbiota, Sex differences, VFAs, Gene expression

## Abstract

**Background:**

The gut microbiota play an important role in maintaining host metabolism, the immune system and health, while sex, genotype, diet and health have specific effects on the composition of the gut microbiota. Therefore, to explore the sex differences in the structure and function of rumen microbiota in Tibetan goats, herein we analyzed sex differences in rumen fermentation parameters, rumen microbiota and the expression of genes related to VFA transport in Tibetan goats.

**Results:**

The results showed that the contents of acetic acid and propionic acid in the rumen of TGM (Tibetan goat male) were significantly higher than those in TGFm (Tibetan goat female) (*P* < 0.05), and total VFAs was significantly higher in TGM than TGFm (*P* < 0.05). Expression of the VFA transport-related genes *DRA*, *AE2*, *MCT-1*, *NHE1*, and *NHE2* in the rumen epithelium of TGFm was significantly higher than that in TGM. Analysis of the composition and structure of the rumen microbiota revealed significant sex differences. At the phylum level, Firmicutes and Bacteroidetes were the dominant phyla in Tibetan goats. In addition, Fibrobacteres and Spirochaetes had significantly greater relative abundances in TGFm than in TGM (*P* < 0.05). At the genus level, the relative abundance of *Fibrobacter*, *Ruminococcus_1* and *Pyramidobacter* was significantly higher in TGFm than in TGM (*P* < 0.05). The functional prediction results showed that replication, recombination and repair, RNA processing and modification were mainly enriched in TGFm (*P* < 0.05).

**Conclusions:**

Correlation analysis revealed significant associations of some rumen microbiota with the fermentation product VFAs and VFA transport-related genes. We concluded that yearling TGM and TGFm have distinct fermentation and metabolism abilities when adapting to the plateau environment, which provides a certain sex reference basis for Tibetan goat adaptation to the plateau environment.

## Introduction

Tibetan goats are an important genetic resource of livestock on the Qinghai-Tibet Plateau, are famous for producing high-quality cashmere and are the dominant animal breed in the development of seasonal animal husbandry in the Qinghai-Tibet Plateau [1]. Living in an alpine region with an altitude of 2500–5000 m, they can adapt to special environmental pressures, such as low oxygen, low temperature, low pressure and strong ultraviolet radiation on the plateau [2]. Through long-term natural selection, Tibetan goats have acquired stable genetic characteristics in physiology, biochemistry and morphology, indicating the unique adaptability of plateau species during long-term evolution [3, 4]. Gut microbes play an important role in the adaptive process of plateau animals. In a previous study, we found that the rumen microbiota of Tibetan sheep underwent significant changes when they adapted to the cold season, and the host genome was significantly correlated with the rumen microbiota and metabolites [5]. Metagenomic sequencing of plateau ruminant yak and Tibetan sheep has shown that rumen microbiota-related genes are significantly enriched in the VFA production pathway, and the energy pathway enriched by VFAs is essential for the adaptive evolution of plateau animals [6]. Liu et al. reported a significant correlation between rumen fermentation parameters and the microbiota composition of yaks, and the composition and function of microbiota can be comprehensively understood through correlation analysis of metabolites [7]. The interaction of rumen microbiota and metabolites has an important impact on the health of the host. Under cold stress conditions, sheep show an improved self-protective mechanism by regulating the relative abundance of rumen microbiota and the concentration of related metabolites [8]. Similarly, heat stress changes the composition of the microbial community, which further significantly affects host energy metabolism of the composition of the rumen microbiota of goats under different temperature and humidity conditions [9].

The composition of animal gut microbiota is affected by a variety of factors, including diet, breed, age, antibiotics, stress, psychology, maternal health, delivery mode, environment and exercise, which all affect the gut microbiota diversity [10]. Chaloner et al. found that the microbiota caused by sex differences exhibits large differences in hormone secretion, energy metabolism, immune response and intestinal parameters [11]. Sex differences play a crucial role in shaping the gut microbiota. Sinha et al. pointed out that the composition of the gut microbiota is significantly different between men and women, and women have a higher microbiota diversity than men [12]. The same result was obtained in a mouse study; the diversity and richness of the gut microbiota in females was higher than that in males [13]. Sex differences in gut microbiota play a key role in the growth and metabolism of the host. Markle et al. found that fecal transplantation from males to females significantly altered the subjects' microbiota and metabolome, and this transplantation increased testosterone levels in mice, alleviated islet inflammation, and protected T1D (type 1 diabetes) development in nonobese diabetic mice [14]. The microbiota can affect innate and adaptive immunity, which indirectly reflects the level of disease resistance caused by sex differences [15]. Therefore, we conducted a comparative analysis of the rumen fermentation function, host-related gene expression and microbiota structure in Tibetan goats of different sexes. Our goal was to understand the changes in the rumen environment and host gene expression in Tibetan goats of different sexes to propose new ideas regarding the interactions among rumen microbiota-VFAs-host genes. Simultaneously, this study also provides a basis for the study of growth and development and feeding patterns in Tibetan goats of different sexes and a reference for the protection and utilization of Tibetan goat germplasm resources.

## Results

### ***Determination results of VFAs, NH***_***3***_***-N and CL***

There were certain differences in VFAs in the rumen fluid of Tibetan goats of different sexes (Table 1). The total VFAs content of TGM was much higher than that of TGFm (*P* < 0.01). Acetic acid and propionic acid were significantly different between sexes, as manifested by significantly higher levels in TGM than in TGFm (*P* < 0.05). The concentrations of isobutyric acid, butyric acid, and isovaleric acid were higher in TGM than in TGFm, but this difference was not significant (*P* > 0.05). There were no significant differences in NH_3_-N content and cellulase (CL) activity between TGFm and TGM (*P* > 0.05).

**Table 1:** Rumen fermentation parameters of Tibetan goat of different sex

Item	TGM	TGFm	***P***
Concentration(mmol/L)			
Acetic acid	38.05 ± 1.30	22.97 ± 0.23	< 0.01
Propionic acid	10.39 ± 1.03	6.85 ± 1.97	0.02
Isobutyric acid	1.05 ± 0.13	0.92 ± 0.12	0.19
Butyric acid	5.26 ± 0.87	4.50 ± 1.08	0.32
Isovaleric acid	1.07 ± 0.07	0.94 ± 0.11	0.82
Valeric acid	1.29 ± 0.50	1.22 ± 0.19	0.09
A/P	4.17 ± 1.90	3.56 ± 0.97	0.81
Total VFAs	52.03 ± 5.08	36.96 ± 2.25	< 0.01
NH_3_-N(mg/100 ml)	39.97 ± 6.40	40.96 ± 2.31	0.78
CL(μg/min/ml)	157.19	154.56	0.14

A/P indicates acetic acid/propionic acid

### Determination of VFA absorption-related gene expression

**Fig. 1:**
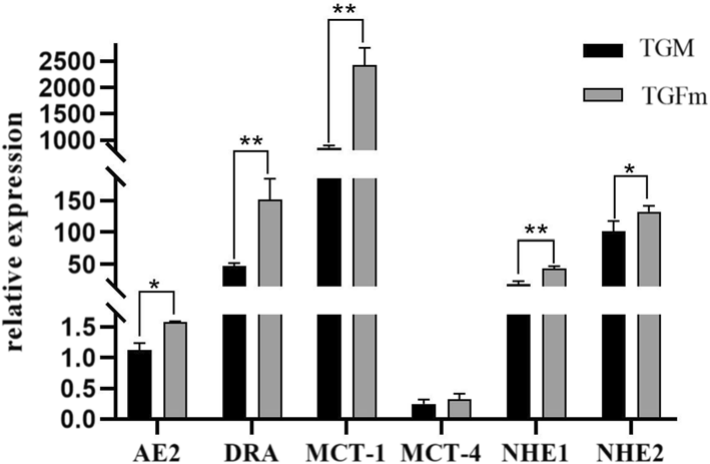
Expression of VFAs transport-related gene. * For *P* < 0.05 and ** for *P* < 0.01

There were differences in the expression of genes related to VFA transport in the rumen epithelium between the TGFm and TGM groups (Fig. 1). The relative expression levels of *AE2*, *DRA*, *NHE1*, *NHE2* and *MCT-1* were significantly higher in TGFm than in TGM (*P* < 0.05). There was no significant difference in the expression of *MCT-4* (*P* > 0.05). Moreover, the expression of *MCT-1*, *AE2*, *DRA*, *MCT-4*, *NHE1* and *NHE2* in TGFm was 2.8 times, 1.4 times, 3.2 times, 1.3 times, 2.3 times and 0.2 times that in TGM, respectively.

### Diversity of rumen microbiota

**Fig. 2:**
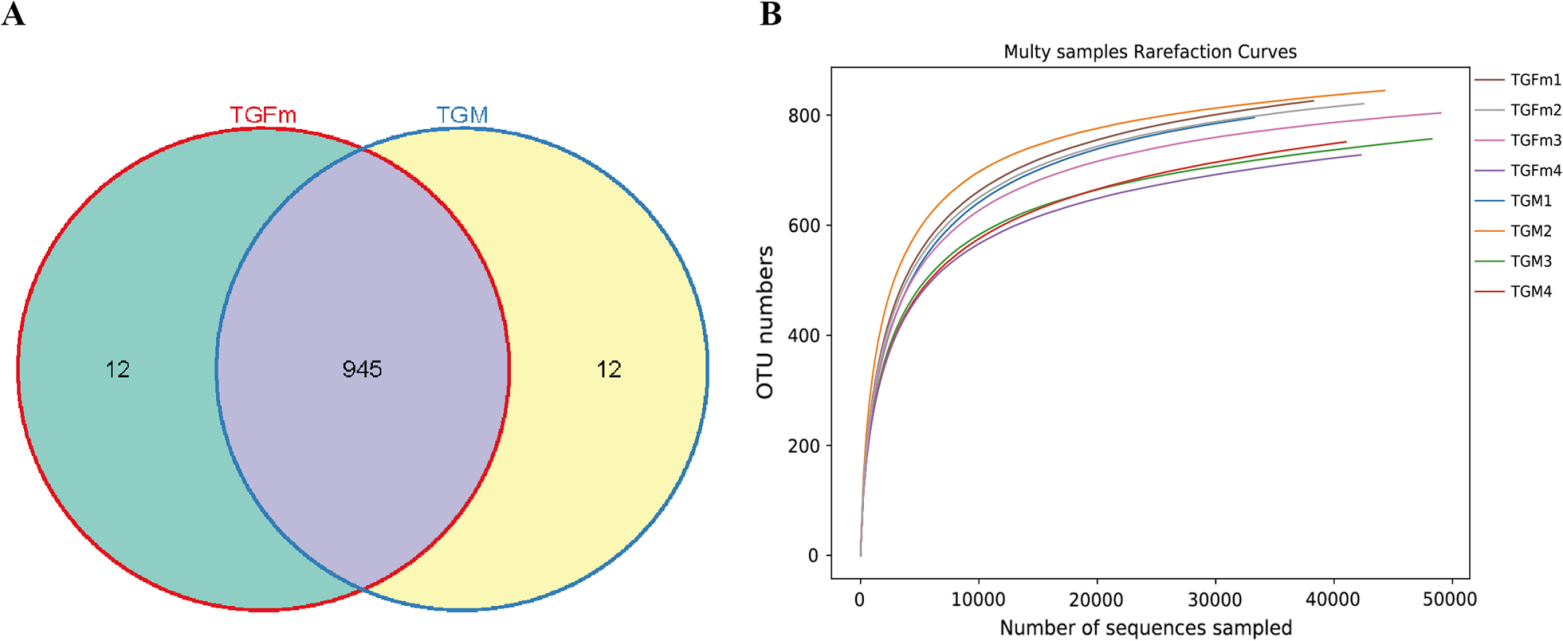
**A** OTU-Venn diagram analysis of TGFm and TGM. **B** Dilution curve analysis

A total of 612,007 pairs of reads were obtained in this study, 610,284 clean reads were generated after tiling and filtering of double-ended reads, and at least 62,730 clean reads were generated for each sample, with an average of 76,286 clean reads produced. Usearch software was used to cluster tags at a similarity level of 97% to obtain the number of OTUs of each sample. A total of 969 OTUs were obtained, including 945 OTUs in TGM and 945 OTUs in TGFm and 12 unique OTUs for each group (Fig. 2A). The dilution curve described the species diversity and species richness of each sample, and the flattening of the curve at 30,000 reads indicated that the sequencing coverage was saturated (Fig. 2B). Alpha diversity analysis (Table 2) showed lower values for TGFm than in TGM regarding ACE and Chao1 indices, but this difference was not significant (*P* > 0.05), while the Shannon and Simpson indices were higher for TGFm than for TGM, although this difference was also not significant (*P* > 0.05).

**Table 2:** Alpha diversity

Index_type	TGFm	TGM	*p*
Shannon	7.47	7.44	0.87
Simpson	0.99	0.98	0.33
ACE	848.31	851.82	0.88
Chao1	868.23	891.64	0.38

### Composition of the rumen microbiota

**Fig. 3:**
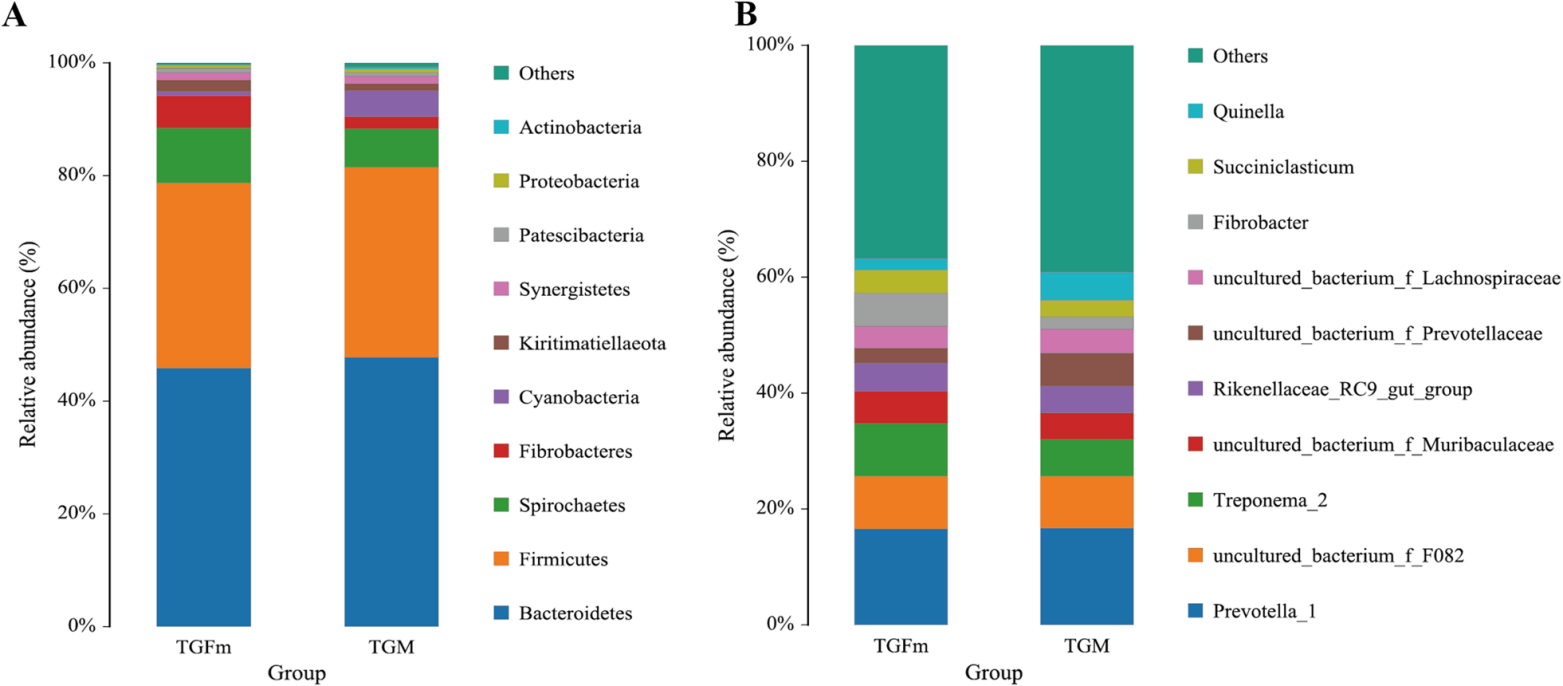
**A** Relative abundance of phylum horizontal species. **B** Relative abundance of genus horizontal species

**Fig. 4:**
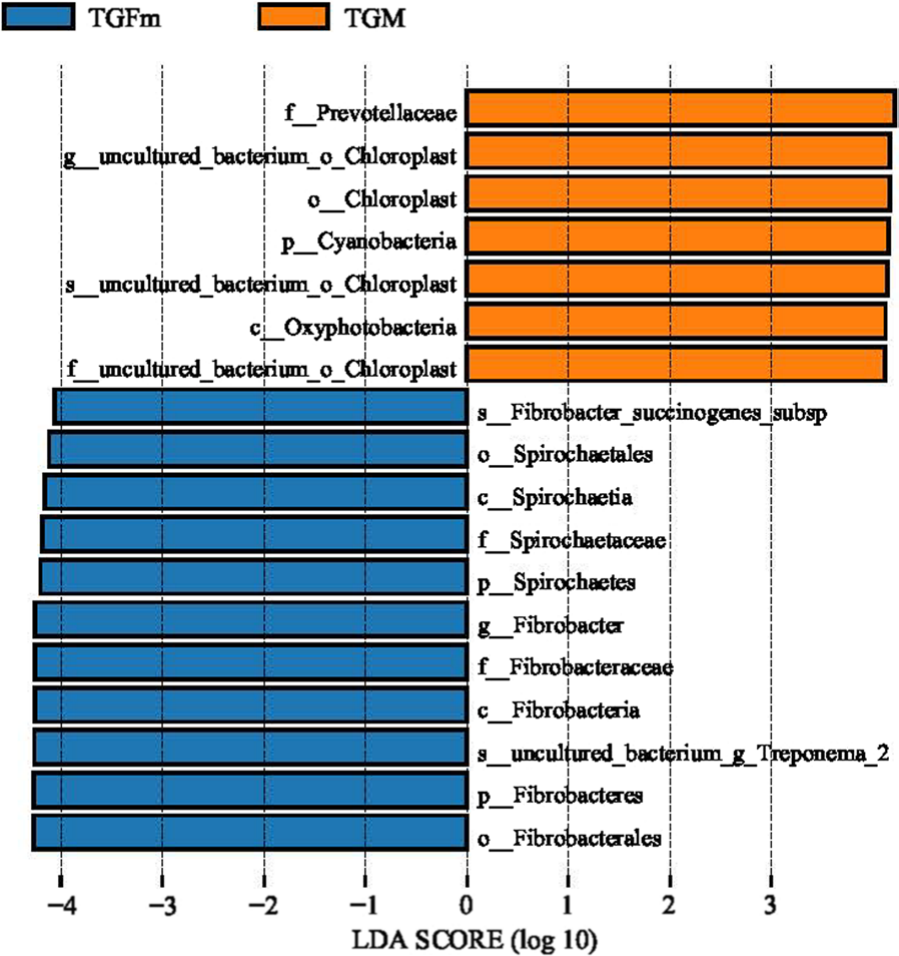
LDA value distribution histogram. LDA value > 4, the length of the bar chart represents the influence of different species

At the taxonomic level, a total of 15 phyla, 14 classes, 31 orders, 55 families, 129 genera, and 156 species were detected in this study. At the phylum level, Bacteroidetes and Firmicutes were the dominant phyla, and the relative abundance of Bacteroidetes in both TGM and TGFm was greater than 45%, while the relative abundance of Firmicutes was greater than 32% (Fig. 3A). The relative abundance of Bacteroidetes and Firmicutes was highest in both TGM and TGFm, accounting for more than 70% of the total abundance. In addition, Fibrobacteres and Spirochaetes had significantly greater relative abundance in TGFm than TGM (*P* < 0.05). The relative abundances of Fibrobacteres and Spirochaetes in TGM accounted for 2.09% and 6.89%, respectively, accounting for 5.71% and 9.76% in TGFm. At the genus level (Fig. 3B), there were 56 genera with relative abundances greater than 0.1%, and *Prevotella_1* was the dominant genus in the rumen of both the TGFm and TGM group. A total of 13 different species were identified among 129 genera (*P* < 0.05). The relative abundance of *Fibrobacter*, *Ruminococcus_1*, *Erysipelotrichaceae_UCG-004*, *Oscillospira* and *Pyramidobacter* was significantly higher in TGFm than in TGM (*P* < 0.05). In addition, the relative abundance of *Lachnospira* and *Ruminococcaceae_NK4A214_group* was significantly higher in TGM than in TGFm (*P* < 0.05). LEfSe analysis of samples between groups (Fig. 4) revealed that there were 7 differential biomarkers (LDA score > 4) for the TGFm and TGM.

### Prediction of rumen microbiota function

A total of 46 KEGG gene families and 25 COG gene families were identified in the 16S rRNA gene sequencing data using PICRUSt software to predict gene function. Among the 25 COG gene families, 2 COG gene families showed significant differences between TGFm and TGM, and the function of replication, recombination and repair, RNA processing and modification (Table 3) showed that TGFm was significantly higher than TGM (*P* < 0.05). Of the 46 KEGG gene families, the vast majority were pathways related to metabolism, of which the largest proportion was the function related to carbohydrate metabolism, followed by amino acid metabolism and energy metabolism. KEGG gene family predictions showed that functions related to amino acid metabolism were increased in TGFm, but this difference was not significant between TGFm and TGM (*P* > 0.05).

**Table 3:** Gene Ontology (GO) and Kyoto Encyclopedia of Genes and Genomes (KEGG) enrichment analysis

Class 1	Class 2	TGFm	TGM	P
Functional prediction of KEGG gene family				
Metabolism	Carbohydrate metabolism	9.40	9.40	0.97
Metabolism	Amino acid metabolism	6.75	6.72	0.45
Metabolism	Energy metabolism	4.09	4.23	0.15
Functional prediction of COG gene family				
Information storage and processing	Replication, recombination and repair	5.75	5.64	0.03
Information storage and processing	RNA processing and modification	0.0040	0.0016	0.02

### Determination of rumen microbiota community density

**Fig. 5:**
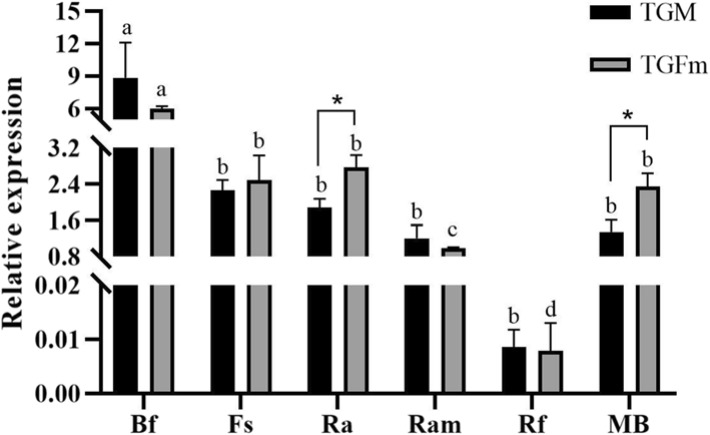
Determination of rumen microbiota density of Tibetan goats of different sexes. Bf indicates Butyrivibrio fibrisolvens, Fs indicates Fibrobacter succinogenes, Ram indicates Ruminobacter amylophilus, Ra indicates Ruminococcus albus, Rf indicates Ruminococcus flavanum, MB indicates Methanogenic bacteria. For different sexes, the same bacterial species marked with * indicates a significant difference between sexes (*P* < 0.05). In the same sex, different bacterial species marked with the same lowercase letters indicate a nonsignificant difference (*P* > 0.05)

Figure 5 shows that there were significant differences in the density of the rumen microbiota of different sexes in Tibetan goats. *Butyrivibrio fibrisolvens* had the highest relative density in Tibetan goats of different sexes, which was significantly higher than that in other microbiota (*P* < 0.05). In the flora of different sexes, the relative densities of *Ruminococcus albus* and *methanogenic bacteria* were higher in the TGFm group than in the TGM group, and the difference was significant between the TGFm and TGM groups (*P* < 0.05). The relative density of *Fibrobacter succinogenes* was higher in TGFm than in TGM, but there was no significant difference (*P* > 0.05). The relative densities of *Butyrivibrio fibrolytica*, *Ruminobacter amylophilus* and *Ruminococcus flavanum* were higher in TGM than TGFm, although this difference was not significant (*P* > 0.05).

### Interactions between rumen microbiota, VFAs and the expression of genes related to transport

**Fig. 6:**
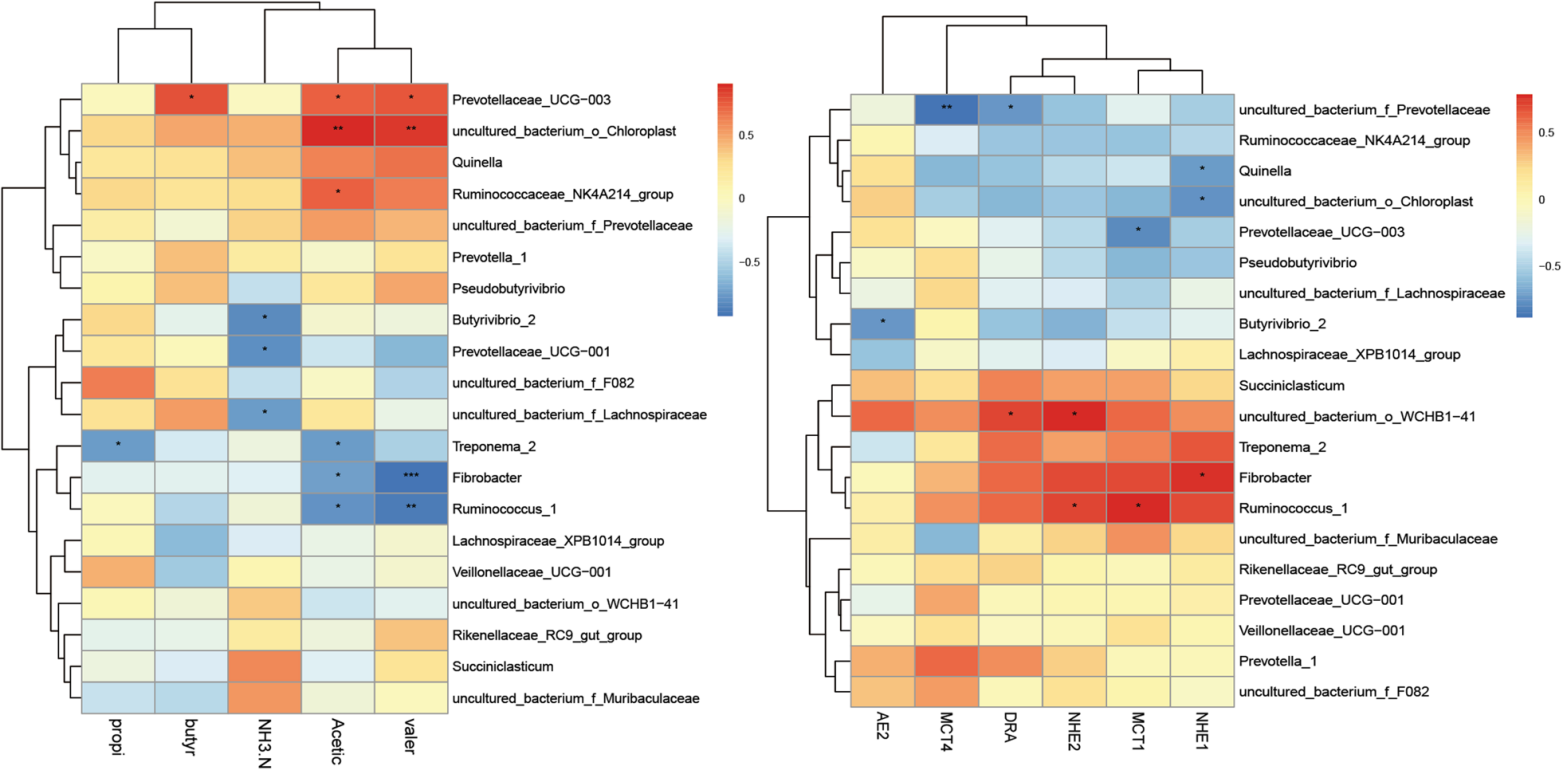
Correlation heat map (* *P* < 0.05, ** *P* < 0.01, *** *P* < 0.001). Acetic indicates acetic acid, butry indicates butyric acid, propi indicates propionic acid, valer indicates valeric acid

As shown in Fig. 6, the correlation heatmap (correlation threshold > 0.5) was constructed between rumen microbiota (top 20 genus level microorganisms with relative abundance) and VFAs and rumen epithelium mRNA expression (2^−∆∆CT^) of Tibetan goats. As shown in Fig. 6, we further found that VFAs were significantly correlated with 6 genus-level microbiota, of which 3 were positively correlated and 3 were negatively correlated (*P* < 0.05). Furthermore, *Ruminococcaceae_NK4A214_group* and *Prevotellaceae_UCG-003* showed significant positive correlations with acetic acid (*P* < 0.05). Moreover, *Treponema_2*, *Fibrobacter* and *Ruminococcus_1* showed significant correlations with acetic acid (*P* < 0.05). *Uncultured_bacterium_f_Lachnospiraceae*, *Butyrivibrio_2* and *Prevotellaceae_UCG-001* were negatively correlated with ammonia nitrogen (*P* < 0.05). In addition, the genes related to VFA transport were correlated with eight genus of microbiota. *Ruminococcus_1* was significantly positively correlated with *NHE2* and *MCT-1* (*P* < 0.05) and negatively correlated with acetic acid (*P* < 0.05). *Fibrobacter* showed a significant positive correlation with *NHE1* (*P* < 0.05) and a significant negative correlation with acetic acid (*P* < 0.05). *Fibrobacter* and *Ruminococcus_1* showed a significant negative correlation with valeric acid (*P* < 0.05). *Prevotellaceae_UCG-003* was significantly negatively correlated with *MCT-1* (*P* < 0.05) and positively correlated with acetic acid (*P* < 0.05).

## Discussion

The Tibetan goat is a special ruminant animal in the Qinghai-Tibet Plateau that provides energy for the body through rumen fermentation of natural herbage. The main components of herbage are cellulose and hemicellulose, while cellulase (CL), as an important enzyme for degrading and digesting fiber substances, can be secreted by fibrolytic bacteria to improve fiber material degradation and digestion [16]. It has been reported that more than 75% of VFAs produced by fermentation are absorbed by the rumen epithelium as the main energy source of ruminants [17]. In this study, we found that the total VFAs in TGM were higher than those in TGFm and that the VFAs produced in the rumen must be transported into the blood circulation through related proteins in the rumen epithelium to provide energy for the body. The monocarboxylate transporter *MCT-1* plays a crucial role in the transport and absorption of VFAs in the rumen epithelial membrane [18]. *MCT-1* and *MCT-4,* as two subtypes with different affinities for VFAs, both play a role in VFA transport in the gastrointestinal tract of ruminants [18]. In addition, Connor et al. observed synergistic effect between the VFA^−^/H^+^ exchange carrier *DRA* and *MCT-1* [18]. This discovery indicated that VFAs were cotransported by multiple transporters in the rumen epithelium. It also found that the expression of the VFA transport genes *MCT-1* and *DRA* increased significantly in the TGFm. We speculated that TGFm transported more VFAs to provide energy and maintain nutritional needs during the process of adapting to the high-altitude environment, resulting in a relatively low concentration of VFAs in the rumen.

Tibetan goats can live in the plateau environment and maintain the reproduction of the population, which is related to the host genome and microbiota called the "second genome" [6]. Studies have reported that dietary fiber is the main energy source for intestinal bacterial fermentation, which can affect estrogen levels and may also shape the gut microbiota [19]. Similarly, intake of fiber also promotes higher levels of dietary microbiota diversity [20]. This present study sequenced the rumen microbiota in Tibetan goats of different sexes and found that the abundance of microbiota was significantly different between TGFm and TGM. It is generally believed that microbiota with high diversity and richness are beneficial to host health, and high microbiota richness is beneficial to microbiota stability [21]. For ruminants, Firmicutes and Bacteroidetes play an important role in degrading fiber and digesting complex carbohydrates [22, 23]. Here, we found that Bacteroidetes and Firmicutes were the main dominant phyla, which is consistent with the results of Wang et al. [24]. Bacteroidetes degrade high-molecular-weight organic matter and improve the innate immune response by enhancing intestinal mucosal barrier function [25, 26]. Firmicutes carry many genes encoding enzymes related to energy metabolism and produce many digestive enzymes to decompose various substances, thereby helping the host digest and absorb nutrients [27]. In the rumen ecosystem, Spirochaetes play a role in the degradation of cellulose, pectin and phytic acid, the utilization of fermentable carbohydrates and the production of volatile fatty acids [28]. In the present study, we found that the relative abundance of spirochaetes in TGFm was significantly higher than that in TGM, which is inconsistent with the results of Hu et al. [29]. At the genus level, the relative abundance of *Lachnospira* was significantly higher in TGM than in TGFm (*P* < 0.05). Jalanka et al. founded *Lachnospira* was associated with the degradation of pectin [30]. Pectin had a strong regulatory effect on the stability of the rumen environment of ruminants, and the rumen flora *Lachnospira* can promote the degradation of pectin, further produce galacturonic acid, inhibit the fermentation of acid lactic acid bacteria, and increase the pH of rumen juice [30, 31]. This result further indicated that Lachnospira in the rumen of TGM could better regulate the rumen environment and prevent acidosis in this study. The genus *Prevotella_1*, a dominant genus of rumen microorganisms, was not significantly different between the TGFm and TGM groups (*P* > 0.05). *Prevotella_1* plays an important role in the degradation and utilization of plant noncellulosic polysaccharides, protein, starch and xylans [32]. In addition, we showed that many cellulolytic bacteria, such as *Ruminococcus_1*, *Fibrobacter* and *Pyramidobacter* were identified. Cellulolytic bacteria are an important bacterial type that play a key role in degrading cellulose materials to produce VFAs in the rumen [33]. Therefore, in this study, the abundances of cellulolytic bacteria such as *Ruminococcus_1*, *Fibrobacter* and *Pyramidobacter* were significantly higher in TGFm than in TGM, indicating that TGFm had a strong fermentation capacity under the harsh plateau environment. TGFm can decompose and ferment more cellulose to produce energy substances VFAs, and these VFAs are further transported by rumen epithelial transporters into the blood for energy, while in the earlier part of this study, the significantly higher expression of VFA transport genes in TGFm further indicated a high metabolic capacity. In addition, the measurement results of the density of the rumen microbiota further tested the microbiota diversity. Studies have found that Ruminococcus albus contains cellulosomes that can adhere to and digest cellulose, and its genome encodes cellulase and hemicellulase [34]. Fibrobacter succinogenes is an anaerobic bacterium naturally colonizing the rumen and cecum of herbivores, where it deconstructs cellulose into cellobiose and glucose, which serve as carbon sources for growth [35]. In this study, *Ruminococcus albus* increased significantly in the TGFm group compared with the TGM group (*P* < 0.05). *Fibrobacter succinogenes* also showed an increase in abundance in TGFm, but this difference was not significant (*P* > 0.05), which further indicated that TGFm had a strong ability to degrade fibrous substances.

Previous studies have reported that the production of propionic acid requires the consumption of rumen hydrogen, which is the main substrate for the growth of methanogens and methanogenesis [36]. The methane produced in the rumen fermentation process is a kind of energy loss to the ruminant host and contributes to the emission of greenhouse gases to the environment [36]. Therefore, we speculated that the significant increase in methanogens in TGFm caused an energy loss, which in turn led to fewer VFAs in the rumen of TGFm. The prediction of microbiota gene function revealed differences in microbiota gene functions in Tibetan goats of different sexes. In the COG gene family, replication, recombination and repair, RNA processing and modification were significantly increased in TGFm. A related study reported that DNA damage caused by inflammation triggered the activation of DNA repair pathways, and the DNA repair mechanism further protected against DNA damage caused by infection and inflammatory diseases and participated in innate and adaptive immunity [37]. Therefore, the results of this study showed that during the process of adapting to a high-altitude environment, TGFm can develop adaptive immunity to maintain normal healthy activities.

**Fig. 7:**
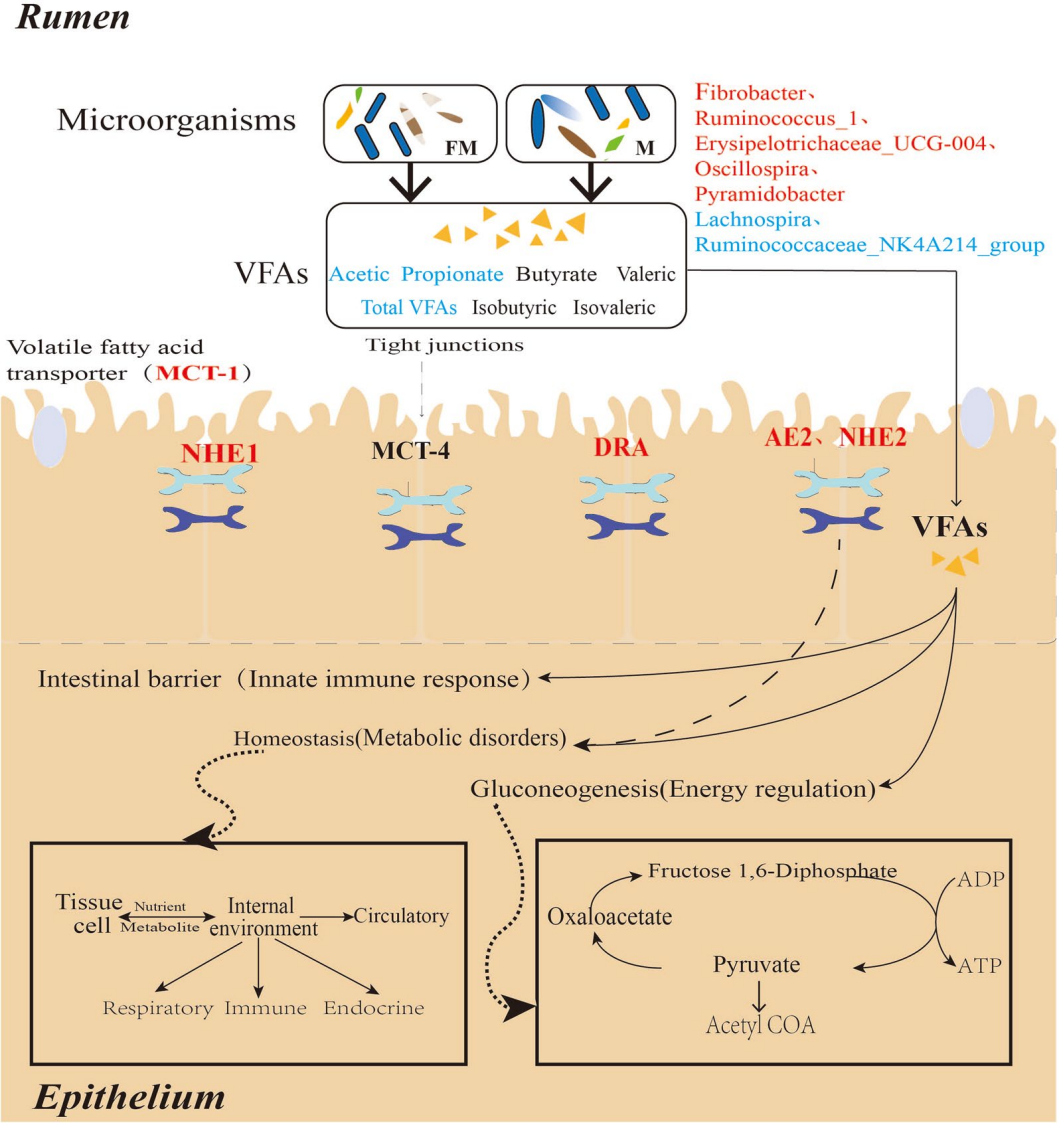
Model of mechanisms for generation and absorption of VFAs in Tibetan goats. Red letters indicate a significant upregulation in TGFm and blue letters indicate a significant upregulation in TGM. Black letters indicate that there is no significant difference between TGFm and TGM

The correlation analysis of rumen microbiota and metabolite VFAs and VFA transport genes showed a certain correlation among them. Previous reports have indicated that *Ruminococcaceae_NK4A214_group* has a significant and positive correlation with glycolysis [38]. Acetic acid significantly increased the muscle expression of key enzymes involved in fatty acid oxidation and glycolytic-to-oxidative fiber-type transformation in exercise-train mice [39]. Acetyl-CoA generated from acetic acid can be preferentially used in the synthesis of citric acid and then participate in citric acid biosynthesis [40]. In this study, correlation analysis showed that *Ruminococcaceae_NK4A214_group* was significantly positively correlated with acetic acid (*P* < 0.05). Therefore, we speculated that *Ruminococcaceae_NK4A214_group* may have a certain influence on the production of acetic acid by affecting the glycolysis pathway, further leading to the significant difference in acetic acid between TGFm and TGM. Acetic acid was significantly negatively correlated with *Ruminococcus_1* (*P* < 0.05), and *MCT-1* was also significantly negatively correlated with *Ruminococcus_1* (*P* < 0.05). Among these VFA transport genes, *MCT-1* transports acetic acid and propionic acid into the blood [18]. This result further explains that when TGFm maintains energy requirements, the VFA transporter can transport relatively more VFAs and then be absorbed and utilized by the rumen epithelium. Thus, the total concentration of rumen VFAs was significantly lower than that of TGM, which further indicated that TGFm had stronger energy metabolism under the traditional grazing condition in the plateau. In addition, the anion exchange protein AE2, located in the basal apical membrane of rumen epithelial cells, plays an important role in regulating homeostasis [41]. In this study, the expression of *AE2* and *NHE2* in TGFm was significantly higher than that in TGM, which might be related to production of more VFAs by the rumen microbiota in TGFm to prevent rumen acidosis and regulate the homeostasis of the rumen. Based on the above results, we found a certain correlation between microbiota-VFAs-host genes (Fig. 7). Under natural grazing conditions in plateaus, the rumen microbiota in Tibetan goats of different sexes showed certain differences, which resulted in different fermentation functions among them. The microbiota produces energy substances, VFAs, by fermenting herbage, which are further transported by rumen epithelial transporters into the blood to supply energy. This interaction mechanism plays an important role in maintaining the nutrient balance between tissue cells and the rumen environment and regulating rumen environment homeostasis.

## Conclusion

This study revealed the differences in the rumen microbiota and rumen fermentation function in Tibetan goats between different sexes. Under the same nutrient level of herbage, total VFAs, propionic acid and acetic acid in TGM were significantly higher than those of TGFm, and the higher expression of VFA transporter genes in TGFm indicated that TGFm had a good efficiency of transporting energy materials. The relative abundance of cellulolytic bacteria in TGFm, such as Firmicutes, Fibrobacteres and Spirochaetes, was significantly higher than that in TGM, which promoted the degradation and fermentation of herbage. Microbiota function prediction revealed that replication, recombination and repair, RNA processing and modification functions were significantly enriched in TGFm, which caused TGFm to participate in innate and adaptive immunity and maintain normal healthy activities. Correlation analysis revealed a significant positive correlation between acetic acid and *Ruminococcaceae_NK4A214_group*. We further inferred that the *Ruminococcaceae_NK4A214_group* might have a certain influence on the production of acetic acid through glycolysis. Based on the above results, the cellulolytic bacteria were significantly more abundant in TGFm than in TGM, causing TGFm to decompose and ferment more cellulose to produce energy substances, such as VFAs. Moreover, VFA transporters could transport can transport relatively more VFAs and then be absorbed and utilized by the rumen epithelium, so the total concentration of rumen VFAs was significantly lower than that in TGM. Thus, yearling female goats have a strong fermentation and metabolism ability in adapting to the special plateau environment compared with male goats, providing an additional basis for the study of adult Tibetan goats.

## Materials and methods

### Experimental animals

Eight healthy Tibetan goats (1 year ± 1 month old) were obtained from a farm in Maqu County (Gansu Province, China) in July 2019, which used local traditional natural grazing management and was located at an altitude of 3700 m. Yearling female goats and Yearling male goats each accounted for half of the population (n = 4).

### Collection of animal samples

Before grazing in the morning, the rumen fluid of Tibetan goats was collected with a gastric tube rumen sampler, and 50 mL of rumen fluid was collected from each goat. The samples were divided into cryopreservation tubes quickly placed into liquid nitrogen tanks and transported to the laboratory for storage at −80 ℃. The samples were used for the determination of 16S rRNA, volatile fatty acids (VFAs) content and ammonia nitrogen (NH_3_-N) content. The animal procedures were approved by relevant departments and in compliance with animal welfare principles. Jugular vein bloodletting was performed (local traditional method), the rumen was removed immediately after death, and a small piece of rumen ventral sac tissue was removed. The contents of the tissue were quickly washed away with normal saline. Subsequently, the rumen epithelial tissue was separated with blunt scissors, rinsed with phosphate-buffered saline (PBS), and stored in liquid nitrogen in cryopreservation tubes, which were transported to the laboratory for storage at -80 ℃ for RNA extraction.

### Collection of forage samples

In the grazing field of experimental animals, sample squares (1 m × 1 m) were used to collect forage samples, and 5 squares (1 m × 1 m) were randomly placed at a distance of more than 10 m. The ground part of the forage samples was collected, and the samples were cut out with scissors. The sample of each square was dried in a laboratory oven at 60 ℃ for 24 h to a constant weight, ground in a mill and passed through a 1 mm sieve for further analysis. The Van Soest method was used to determine the concentration of acid detergent fiber (ADF) and neutral detergent fiber (NDF) in 5 samples [42]. The AOAC method was used to determine dry matter (DM), crude protein (CP), crude fatty ether extract (EE), crude ash (Ash), calcium and phosphorus [43]. The measurement results are shown in Table 4.

**Table 4:** Summary nutrient determination results of plateau forage (%)

CP	EE	DM	Ash	NDF	ADF	Ca	P
10.64	4.07	5.39	6.95	54.95	32.15	0.84	1.14

### Determination of rumen fermentation parameters

VFAs were determined using a Shimadzu (GC-2010 PLUS) gas chromatograph with an internal standard method, and the internal standard was 2-ethylbutyric acid (2 EB). The determination was performed on an AT-FFAP capillary column (50 m × 0.32 mm × 0.25 μm). The column temperature was maintained at 60 ℃ for 1 min, then increased to 115 ℃ at 5 ℃/min without reservation, and then increased to 180 ℃ at 15 ℃/min. The detector temperature was 260℃, and the injector temperature was 250 ℃. The content of NH_3_-N in rumen fluid was determined by spectrophotometer colorimetry (spectrophotometer UV). A cellulase (CL) activity assay kit (Suzhou Keming Biotechnology Company Limited, China) was used to determine the activity of cellulase in rumen fluid.

### DNA extraction, high-throughput sequencing and colony density determination

The bacterial DNA was isolated from each rumen sample using an MN NucleoSpin 96 Soil kit (Macherey–Nagel, Germany). The bacterial V3-V4 region of 16S rRNA genes in the total DNA was amplified using specific primers (forward primer 338F: 5′-ACTCCTACGGGAGgCAGCA-3′ and reverse primer 806R: 5′-GGACTACHVGGGTWTCTAAT-3′). The rumen microbiota was sequenced using a two-step library construction method, and amplified products were sequenced on Illumina MiSeq 2500 (Illumina, San Diego, CA, USA) platform. Using rumen microbiota DNA as a template, methanogens, protein-degrading bacteria and fiber-degrading bacteria were selected for population density determination and analysis (see Table 5 for sequence information). Bacteria were used as an internal reference, and bacterial primers referred to the study reported by Muyze et al. [44]. The sequencing data were deposited into the Sequence Read Archive (SRA) of NCBI (Accession Nos. SRR16930752–SRR16930759).

**Table 5:** Primer sequences of microbiota

Gene	Primer(5′–3′)	Length	Annealing temperature (℃)	Accession number
Bacterium	F:CCTACGGGAGGCAGCAG	181 bp	60	*
	R:TTACCGCGGCTGCTGG			
Rf	F:TATCTTAGTGGCGGACGGGT	157 bp	60	MT356193.1
	R:TCTAATCAGACGCGAGCCCA			
Fs	F:GATGAGCTTGCGTCCGATT	110 bp	60	EU606019.1
	R:ATTCCCTACTGCTGCCTCC			
Ra	F:GGGCTTAACCCCTGAACTGC	114 bp	60	X85098.1
	R:TCGCCACTGATGTTCCTCCT			
Ram	F:GGGGACAACACCTGGAAACG	124 bp	60	Y15992.1
	R:CTTGGTAGGCCGTTACCCCA			
Bf	F:CCTGACTAAGAAGCACCGGC	107 bp	60	U41167.1
	R:GTAAAACCGCCTACGCTCCC			
MB	F:TCTGTACGGGTTGTGAGAGCA	106 bp	60	KP752401.1
	R:CGCGATTTCTCACATTGCGG			

### Determination of mRNA expression in rumen epithelial tissue

Total RNA was extracted from rumen epithelial tissues of Tibetan goats using the TRIzol reagent method (TransGen). The concentration and purity of RNA were determined using an ultramicro spectrophotometer (Thermo Nano drop-2000). A reverse transcription kit (HiScript® II Q RT SuperMix for qPCR; Nanjing, China) was used to synthesize cDNA. Specific primers were designed using Primer 5.0 software (Table 6). An Applied Biosystems Q6 quantitative PCR instrument was used to quantify the fluorescence of the rumen epithelium-related genes and the internal reference gene. The resulting data were analyzed using the 2^−∆∆CT^ method and β-actin as the internal reference gene for correction. Reaction conditions: 95 °C predenaturation for 30 s; cyclic reaction at 95 °C for 10 s, 60 °C for 30 s, 40 cycles; dissolution curve (95 °C for 15 s, 60 °C for 60 s, 95 °C for 15 s).

**Table 6:** Primer sequences of fatty acid-related genes

Gene	Primer (5′–3′)	Length	Annealing temperature (°C)	Accession number
*β-actin*	F:AGCCTTCCTTCCTGGGCATGGA	113 bp	60	NM_001009784.3
	R:GGACAGCACCGTGTTGGCGTAGA			
*DRA*	F:TGTGGCGGCTTCCAGAATTT	167 bp	60	NM_001280717.1
	R:CACAGGCTTGTTTGGGAGCA			
*MCT1*	F:GGACTGTGTCATCTGGCAGC	134 bp	60	XM_004002335.4
	R:TGGGGTCCAACAAGGTCCAT			
*AE2*	F:AAGATCCCTGAGAACGCCGA	152 bp	60	XM_027969012.1
	R:AGCAGAAAGAGGAAGCGCAC			
*NHE2*	F:TTCTTTGTCGTGGGGATCGG	180 bp	60	XM_027967037.1
	R:CGTGATTGCCATGATGCCTG			
*MCT4*	F:ACGGCTCAGCCTTAGTAAACTTC	144 bp	60	NC_0402252.1
	R:AATGGAGTTGTGCGAGTTGGT			
*NHE1*	F:GCTTCTTCGTGGTGTCCCTG	174 bp	60	XM_004005085.4
	R:CCATGATGCCTGACAGGTGG			

### Bioinformatics analysis

Quality assessment of the original sequencing data: the original sequencing reads are denoised, paired-end spliced (FLASH, version 1.2.11), quality filtered (Trimmomatic, version0.33), and chimera removed (UCHIME, version 8.1). The Usearch software (Version 10.0) was used to cluster the high quality effective Tags with the 2013 Greengenes (version 13.8) ribosome database at 97% similarity level. Operational taxonomic units (OTUs) was filtered with 0.005% of all sequences sequenced as the threshold, and species annotation and taxonomic analysis were performed on OTU based on Silva (Bacteria 16S) database [45].

Alpha diversity analysis was performed on OTU analysis results by Mothur (version V.1.30), and the Rarefaction Curve and Shannon Index Dilution Curve were plotted. Different species between groups were obtained by LefSe analysis.

Finally, PICRUST software was used to compare the species composition information obtained from 16S sequencing data to analyze the functional differences among different groups. KEGG (Kyoto Encyclopedia of Genes and Genomes) difference analysis was performed to observe the differences and variations in metabolic pathways of functional genes in microbial communities among different groups of samples. COG (Clusters of Orthologous Groups of proteins) analysis predicts the differences and changes of prokaryotic functions between different groups.

### Statistical data analysis

The independent sample T test in SPSS software (version 24.0, SPSS Inc.) was used to analyze differences in rumen fermentation parameters (VFAs), CL activity and Alpha diversity index (Ace index, Chao1 index, Shannon index and Simpson index) of Tibetan goat of different sex. Linear discriminant analysis (LDA) effect size (LEfSe) method was used to evaluate the differences of microbial communities, and the LDA score threshold was 4. The analysis data were all expressed as Mean ± SD, and the statistical significance level is *P* < 0.05. Spearman was used for correlation test.

## Data Availability

Raw sequence data associated with is being uploaded to NCBI.

## Abbreviations

CL: Cellulase
VFAs: Volatile fatty acid
NH_3_-N: Ammonia nitrogen

## References

[1] Jin M, Lu J, Fei X, Lu Z, Quan K, Liu Y, Chu M, Di R, Wei C, Wang H (2020). Selection signatures analysis reveals genes associated with high-altitude adaptation in Tibetan goats from Nagqu. Tibet Animals (Basel).

[2] Thompson LG, Yao T, Mosley-Thompson E, Davis ME, Henderson KA, Lin P (2000). A high-resolution millennial record of the south Asian monsoon from himalayan ice cores. Science.

[3] Deng J, Feng J, Li L, Zhong T, Wang L, Guo J, Ba G, Song T, Zhang H (2018). Polymorphisms, differentiation, and phylogeny of 10 Tibetan goat populations inferred from mitochondrial D-loop sequences. Mitochondrial DNA A DNA Mapp Seq Anal.

[4] Guo J, Tao H, Li P, Li L, Zhong T, Wang L, Ma J, Chen X, Song T, Zhang H (2018). Whole-genome sequencing reveals selection signatures associated with important traits in six goat breeds. Sci Rep.

[5] Liu X, Sha Y, Dingkao R, Zhang W, Lv W, Wei H, Shi H, Hu J, Wang J, Li S, Hao Z, Luo Y (2020). Interactions between rumen microbes, VFAs, and host genes regulate nutrient absorption and epithelial barrier function during cold season nutritional stress in Tibetan sheep. Front Microbiol.

[6] Zhang Z, Xu D, Wang L, Hao J, Wang J, Zhou X, Wang W, Qiu Q, Huang X, Zhou J, Long R, Zhao F, Shi P (2016). Convergent evolution of rumen microbiomes in high-altitude mammals. Curr Biol.

[7] Liu C, Wu H, Liu S, Chai S, Meng Q, Zhou Z (2019). Dynamic Alterations in Yak Rumen bacteria community and metabolome characteristics in response to feed type. Front Microbiol.

[8] Guo H, Zhou G, Tian G, Liu Y, Dong N, Li L, Zhang S, Chai H, Chen Y, Yang Y (2021). Changes in Rumen microbiota affect metabolites, immune responses and antioxidant enzyme activities of sheep under cold stimulation. Animals.

[9] Zhong S, Ding Y, Wang Y, Zhou G, Guo H, Chen Y, Yang Y (2019). Temperature and humidity index (THI)-induced rumen bacterial community changes in goats. Appl Microbiol Biotechnol.

[10] Osadchiy V, Martin CR, Mayer EA (2019). The gut-brain axis and the microbiome: mechanisms and clinical implications. Clin Gastroenterol Hepatol.

[11] Chaloner A, Greenwood-Van MB (2013). Sexually dimorphic effects of unpredictable early life adversity on visceral pain behavior in a rodent model. J Pain.

[12] Sinha T, Vich Vila A, Garmaeva S, Jankipersadsing SA, Imhann F, Collij V, Bonder MJ, Jiang X, Gurry T, Alm EJ, D'Amato M, Weersma RK, Scherjon S, Wijmenga C, Fu J, Kurilshikov A, Zhernakova A (2019). Analysis of 1135 gut metagenomes identifies sex-specific resistome profiles. Gut Microbes.

[13] Elderman M, Hugenholtz F, Belzer C, Boekschoten M, van Beek A, de Haan B, Savelkoul H, de Vos P, Faas M (2018). Sex and strain dependent differences in mucosal immunology and microbiota composition in mice. Biol Sex Differ.

[14] Markle JG, Frank DN, Mortin-Toth S, Robertson CE, Feazel LM, Rolle-Kampczyk U, von Bergen M, McCoy KD, Macpherson AJ, Danska JS (2013). Sex differences in the gut microbiome drive hormone-dependent regulation of autoimmunity. Science.

[15] Rizzetto L, Fava F, Tuohy KM, Selmi C (2018). Connecting the immune system, systemic chronic inflammation and the gut microbiome: the role of sex. J Autoimmun.

[16] Bhat AH, Khan I, Usmani MA, Umapathi R, Al-Kindy SMZ (2019). Cellulose an ageless renewable green nanomaterial for medical applications: an overview of ionic liquids in extraction, separation and dissolution of cellulose. Int J Biol Macromol.

[17] Russell JB, Rychlik JL (2001). Factors that alter rumen microbial ecology. Science.

[18] Connor EE, Li RW, Baldwin RL, Li C (2010). Gene expression in the digestive tissues of ruminants and their relationships with feeding and digestive processes. Animal.

[19] Dominianni C, Sinha R, Goedert JJ, Pei Z, Yang L, Hayes RB, Ahn J (2015). Sex, body mass index, and dietary fiber intake influence the human gut microbiome. PLoS ONE..

[20] Fernandes KA, Kittelmann S, Rogers CW, Gee EK, Bolwell CF, Bermingham EN, Thomas DG (2014). Faecal microbiota of forage-fed horses in New Zealand and the population dynamics of microbial communities following dietary change. PLoS ONE.

[21] Tap J, Furet JP, Bensaada M, Philippe C, Roth H, Rabot S, Lakhdari O, Lombard V, Henrissat B, Corthier G, Fontaine E, Doré J, Leclerc M (2015). Gut microbiota richness promotes its stability upon increased dietary fibre intake in healthy adults. Environ Microbiol.

[22] Thoetkiattikul H, Mhuantong W, Laothanachareon T, Tangphatsornruang S, Pattarajinda V, Eurwilaichitr L, Champreda V (2013). Comparative analysis of microbial profiles in cow rumen fed with different dietary fiber by tagged 16S rRNA gene pyrosequencing. Curr Microbiol.

[23] Spence C, Wells WG, Smith CJ (2006). Characterization of the primary starch utilization operon in the obligate anaerobe Bacteroides fragilis: Regulation by carbon source and oxygen. J Bacteriol.

[24] Wang X, Zhang Y, Wen Q, Wang Y, Wang Z, Tan Z, Wu K (2020). Sex differences in intestinal microbial composition and function of Hainan special wild boar. Animals (Basel).

[25] Thomas F, Hehemann JH, Rebuffet E, Czjzek M, Michel G (2011). Environmental and gut bacteroidetes: the food connection. Front Microbiol.

[26] Magrone T, Jirillo E (2013). The interplay between the gut immune system and microbiota in health and disease: nutraceutical intervention for restoring intestinal homeostasis. Curr Pharm Des.

[27] Kaakoush NO (2015). Insights into the role of erysipelotrichaceae in the human host. Front Cell Infect Microbiol.

[28] Hess M, Sczyrba A, Egan R, Kim TW, Chokhawala H, Schroth G, Luo S, Clark DS, Chen F, Zhang T, Mackie RI, Pennacchio LA, Tringe SG, Visel A, Woyke T, Wang Z, Rubin EM (2011). Metagenomic discovery of biomass-degrading genes and genomes from cow rumen. Science.

[29] Hu D, Chao Y, Li Y, Peng X, Wang C, Wang Z, Zhang D, Li K (2021). Effect of gender bias on equine fecal microbiota. J Equine Vet Sci.

[30] Jalanka J, Major G, Murray K, Singh G, Nowak A, Kurtz C, Silos-Santiago I, Johnston JM, de Vos WM, Spiller R (2019). The effect of psyllium husk on intestinal microbiota in constipated patients and healthy controls. Int J Mol Sci.

[31] Canani RB, Costanzo MD, Leone L, Pedata M, Meli R, Calignano A (2011). Potential beneficial effects of butyrate in intestinal and extraintestinal diseases. World J Gastroenterol.

[32] Liu H, Xu T, Xu S (2019). Effect of dietary concentrate to forage ratio on growth performance, rumen fermentation and bacterial diversity of Tibetan sheep under barn feeding on the Qinghai-Tibetan plateau. PeerJ.

[33] Pan X, Xue F, Nan X, Tang Z, Wang K, Beckers Y, Jiang L, Xiong B (2017). Illumina sequencing approach to characterize thiamine metabolism related bacteria and the impacts of thiamine supplementation on ruminal microbiota in dairy cows fed high-grain diets. Front Microbiol.

[34] Suen G, Stevenson DM, Bruce DC, Chertkov O, Copeland A, Cheng JF (2011). Complete genome of the cellulolytic ruminal bacterium *Ruminococcus albus* 7. J Bacteriol.

[35] Arntzen MØ, Várnai A, Mackie RI, Eijsink VGH, Pope PB (2017). Outer membrane vesicles from Fibrobacter succinogenes S85 contain an array of carbohydrate-active enzymes with versatile polysaccharide-degrading capacity. Environ Microbiol.

[36] Romero-Huelva M, Ramos-Morales E, Molina-Alcaide E (2012). Nutrient utilization, ruminal fermentation, microbial abundances, and milk yield and composition in dairy goats fed diets including tomato and cucumber waste fruits. J Dairy Sci.

[37] Fontes FL, Pinheiro DM, Oliveira AH, Oliveira RK, Lajus TB, Agnez-Lima LF (2015). Role of DNA repair in host immune response and inflammation. Mutat Res Rev Mutat Res.

[38] Pacífico C, Petri RM, Ricci S, Mickdam E, Wetzels SU, Neubauer V, Zebeli Q (2021). Unveiling the bovine epimural microbiota composition and putative function. Microorganisms.

[39] Pan JH, Kim JH, Kim HM, Lee ES, Shin DH, Kim S, Shin M, Kim SH, Lee JH, Kim YJ (2015). Acetic acid enhances endurance capacity of exercise-trained mice by increasing skeletal muscle oxidative properties. Biosci Biotechnol Biochem.

[40] Des Rosiers C, David F, Garneau M, Brunengraber H (1991). Nonhomogeneous labeling of liver mitochondrial acetyl-CoA. J Biol Chem.

[41] Bilk S, Huhn K, Honscha KU, Pfannkuche H, Gäbel G (2005). Bicarbonate exporting transporters in the ovine ruminal epithelium. J Comp Physiol B.

[42] Van Soest PJ, Robertson JB, Lewis BA (1991). Methods for dietary fiber, neutral detergent fiber, and nonstarch polysaccharides in relation to animal nutrition. J Dairy Sci.

[43] Helrich K, Helrich K. Official Methods of Analysis of the AOAC.1990.

[44] Muyzer G, de Waal EC, Uitterlinden AG (1993). Profiling of complex microbial populations by denaturing gradient gel electrophoresis analysis of polymerase chain reaction-amplified genes coding for 16S rRNA. Appl Environ Microbiol.

[45] Bokulich NA, Subramanian S, Faith JJ, Gevers D, Gordon JI, Knight R, Mills DA, Caporaso JG (2013). Quality-filtering vastly improves diversity estimates from Illumina amplicon sequencing. Nat Methods.

